# Efficacy of Enfortumab Vedotin After Platinum Chemotherapy and Pembrolizumab in Metastatic Urothelial Carcinoma: A Multicenter Real-World Analysis of the ARON-2EV Cohort

**DOI:** 10.1016/j.euros.2025.10.010

**Published:** 2025-11-03

**Authors:** Ondrej Fiala, Francesco Grillone, Kazutoshi Fujita, Enrique Grande, Patrizia Giannatempo, Tarek Taha, Zin W. Myint, Thomas Büttner, Alfonso Gómez de Liaño, Ravindran Kanesvaran, Gaetano Facchini, Akihiro Yano, Luigi Formisano, Alexandr Poprach, Vincenza Conteduca, Alina Pirshtuk, Hana Studentova, Jindrich Kopecky, Enrico Sammarco, Augusto Mota, Lorena Incorvaia, Cecilia Nasso, Michele Maffezzoli, Aruni Ghose, Andrey Soares, Sebastiano Buti, Fernando Sabino Marques Monteiro, Francesco Massari, Shilpa Gupta, Joaquim Bellmunt, Giuseppe Luigi Banna, Matteo Santoni

**Affiliations:** a Department of Oncology and Radiotherapeutics, Faculty of Medicine and University Hospital Pilsen, Charles University, Pilsen, Czechia; b Biomedical Center, Faculty of Medicine, Charles University, Pilsen, Czechia; c SOC Oncologia PO Pugliese-Ciaccio Azienda Ospedaliera Universitaria Renato Dulbecco, Catanzaro, Italy; d Department of Urology, Kindai University, Osaka, Japan; e Department of Medical Oncology, MD Anderson Cancer Center Madrid, Madrid, Spain; f Dipartimento di Oncologia Medica, Fondazione IRCCS Istituto Nazionale dei Tumori, Milan, Italy; g Royal Marsden NHS Foundation Trust, London, UK; h Division of Medical Oncology, Department of Internal Medicine, Markey Cancer Center, University of Kentucky, Lexington, KY, USA; i Department of Urology, University Hospital Bonn, Bonn, Germany; j Medical Oncology Department, CHU Insular-Materno Infantil, Las Palmas, Spain; k Division of Medical Oncology, National Cancer Centre Singapore, Singapore; l Oncology Operative Unit, Santa Maria delle Grazie Hospital, Pozzuoli, Italy; m Department of Urology, Saitama Medical Center, Saitama Medical University, Kawagoe, Saitama, Japan; n Department of Medicine and Surgery, Federico II University, Naples, Italy; o Masaryk Memorial Cancer Institute, Brno, Czechia; p Faculty of Medicine, Masaryk University, Brno, Czechia; q Unit of Medical Oncology and Biomolecular Therapy, Department of Medical and Surgical Sciences, University of Foggia, Policlinico Riuniti, Foggia, Italy; r Department of Oncology, Second Faculty of Medicine, Charles University and University Hospital Motol, Prague, Czechia; s Department of Oncology, Palacký University Medical School and Teaching Hospital, Olomouc, Czechia; t Department of Oncology, University Hospital Hradec Králové, Hradec Králové, Czechia; u Medical Oncology Unit, Livorno Hospital, Azienda Toscana Nord Ovest, Livorno, Italy; v Clínica Assistência Multidisciplinar em Oncologia, Salvador, Brazil; w Department of Precision Medicine in Medical, Surgical and Critical Care, Section of Medical Oncology, University of Palermo, Palermo, Italy; x Division of Oncology, S. Corona Hospital, Pietra Ligure, Italy; y Department of Oncology, Portsmouth Hospitals University NHS Trust, Portsmouth, UK; z Barts Cancer Centre, St .Bartholomew’s Hospital, Barts Health NHS Trust, London, UK; aa Oncology Unit, Hospital Israelita Albert Einstein, São Paulo, Brazil; bb Latin American Cooperative Oncology Group, Porto Alegre, Brazil; cc Medical Oncology Unit, University Hospital of Parma, Parma, Italy; dd Department of Medicine and Surgery, University of Parma, Parma, Italy; ee Oncology and Hematology Department, Hospital Sírio Libanês, Brasília, Brazil; ff Medical Oncology, IRCCS Azienda Ospedaliero-Universitaria di Bologna, Bologna, Italy; gg Department of Medical and Surgical Sciences, University of Bologna, Bologna, Italy; hh Taussig Cancer Institute, Cleveland Clinic, Cleveland, OH, USA; ii Dana Farber Cancer Institute, Harvard Medical School, Boston, MA, USA; jj School of Pharmacy and Biomedical Sciences, University of Portsmouth, Portsmouth, UK; kk Medical Oncology Unit, Macerata Hospital, Macerata, Italy

**Keywords:** Urothelial cancer, Enfortumab vedotin, Pembrolizumab, ARON-2EV study, Clinical trial, NCT05290038

## Abstract

**Background and objective:**

Enfortumab vedotin (EV) has demonstrated efficacy in metastatic urothelial carcinoma (mUC) following treatment with platinum-based chemotherapy (PBC) and PD-1/PD-L1 inhibitors, including pembrolizumab (PEM). Our aim was to assess real-world clinical outcomes of EV in patients with mUC previously treated with PBC followed by pembrolizumab (PBC/PEM).

**Methods:**

ARON-2EV is an international, multicenter, retrospective study examining the real-world use of EV in patients with mUC. This analysis included 401 patients with mUC treated with EV following PBC/PEM. Primary endpoints were overall survival (OS), time on treatment (ToT), and the overall response rate (ORR). Secondary objectives included evaluation of clinical factors associated with outcomes and exploration of the impact of prior PEM outcomes. Statistical methods included Kaplan-Meier estimates, log-rank tests, Fisher’s exact and χ^2^ tests, Pearson’s correlation coefficients, and Cox analyses.

**Key findings and limitations:**

Median OS was 12.3 mo (95% confidence interval [CI] 9.9–14.3), median ToT was 10.4 mo (95% CI 9.0–12.2), and the ORR was 45%. Prior response to PEM was significantly correlated with subsequent outcomes on EV. In the group who experienced progressive disease (PD) on prior PEM, the ORR was 35%. Major limitations are related to the retrospective study design.

**Conclusions:**

EV has consistent real-world efficacy in patients with mUC following sequential treatment with PBC/PEM. These results support the utility of EV in diverse clinical scenarios and suggest an association between prior PEM outcomes and subsequent EV efficacy.

**Patient summary:**

The ARON-2EV project is collecting real-world data for patients with metastatic cancer of the urinary tract who are being treated with a drug called enfortumab vedotin (EV) in multiple centers worldwide. Our results show that EV has consistent efficacy in these patients after previous treatment with platinum-based chemotherapy followed by pembrolizumab. The results also suggest an association between a patient’s response to pembrolizumab and their subsequent response to EV.

## Take Home Message

Our results support the real-world efficacy of enfortumab vedotin (EV) in patients with metastatic urothelial carcinoma previously treated with platinum-based chemotherapy followed by pembrolizumab. We observed an association between prior pembrolizumab outcomes and subsequent EV efficacy. This suggests that prior immunotherapy outcomes could serve as practical clinical indicators of potential benefits from subsequent EV treatment.

## 1 Introduction

Urothelial carcinoma (UC) is a prevalent malignancy of the genitourinary tract that is characterized by significant clinical and morphological heterogeneity. It can arise in the renal pelvis, ureter, or bladder, with bladder being the most frequent primary site. It has been estimated that the global incidence of bladder cancer in 2022 was 614 298 new cases, resulting in 220 596 deaths [1]. In terms of histological subtypes, pure UC is found in ∼75% of cases, while variant histologies, which represent aberrant differentiation, are seen in 25% of cases [2]. Approximately 25% of patients with UC present with metastatic disease at diagnosis, which is associated with poor prognosis and a 5-yr survival rate of 7.7%. [3]. Owing to its aggressive nature and both intrinsic and acquired resistance to chemotherapy, treatment of metastatic UC (mUC) remains a significant challenge in urological oncology [4,5].

First-line treatment for patients with mUC currently includes enfortumab vedotin (EV) combined with pembrolizumab or platinum-based chemoimmunotherapy (concomitant or in switch maintenance), both of which are considered standard systemic approaches [6]. Immune checkpoint inhibitors (ICIs) targeting the PD-1/PD-L1 axis were initially introduced as a second-line treatment for patients with disease progression following frontline platinum-based chemotherapy (PBC). At that stage, three ICIs were approved for use: pembrolizumab (PEM) [7], atezolizumab [8], and nivolumab [9]. Subsequently, switch maintenance therapy with avelumab became a standard option for patients achieving an objective response or stable disease after PBC [10]. More recently, the combination of nivolumab and cisplatin-based chemotherapy has emerged as a treatment strategy for cisplatin-eligible patients [11].

Antibody-drug conjugates (ADCs) are innovative anticancer therapies with significant potential across a range of malignancies, including mUC. EV is an ADC that combines a fully human IgG1-κ antibody targeting NECTIN4, a cell-surface adhesion protein expressed on UC cells, with the microtubule-disrupting agent monomethyl auristatin E. This conjugate is linked via a protease-sensitive maleimidocaproyl-valine-citrulline linker that ensures targeted delivery of the cytotoxic agent to tumor cells [12,13].

The efficacy and safety of EV in patients with mUC who have received prior treatments have been established via prospective clinical trials, including the nonrandomized phase 2 EV-201 study [14] and the randomized phase 3 EV-301 trial [15]. In EV-301, EV monotherapy was associated with a significant improvement in both progression-free survival (PFS) and overall survival (OS) in comparison to single-agent chemotherapy in patients with mUC who had previously been treated with PBC and PD-1/PD-L1 inhibitors, which ultimate led to US Food and Drug Administration approval of EV for this patient population [16].

ARON-2EV (NCT05290038) was designed as an international, retrospective, multicenter study to collect global real-world data on EV use in patients with mUC who progressed after previous PBC and anti-PD-1/PD-L1 inhibitor therapy. The present analysis of ARON-2EV specifically evaluated the efficacy of EV in the subgroup of patients with disease progression after PBC followed by PEM (PBC/PEM).

## 2 Patients and methods

### 2.1 Study design and patient population

ARON-2EV was conducted across 51 oncology centers in 24 countries (Supplementary Fig. 1). Eligible participants were adults (≥18 yr) with a confirmed diagnosis of mUC according to cytology and/or histology. Data for patients who received EV between January 1, 2016, and November 30, 2024 after previous PBC/PEM treatment were analyzed. Clinical data were collected from medical reports at each participating center. EV was administered intravenously as a single agent according to the approved regimen (1.25 mg/kg on days 1, 8, and 15 of each 28-d cycle). Treatment with EV continued until disease progression, unacceptable toxicity, or patient withdrawal. At each center, standard procedures for physical examinations, laboratory tests, and imaging methods, including computed tomography and magnetic resonance imaging scans, were followed according to current clinical guidelines.

### 2.2 Study objectives

The primary objective was to evaluate clinical efficacy outcomes for patients with mUC receiving EV following PBC/PEM. The primary endpoints were OS, time on treatment (ToT), and the overall response rate (ORR). OS was defined as time from the start of EV treatment until death from any cause. ToT was defined as time from the start of therapy until permanent treatment discontinuation for any cause, including disease progression, toxicity, patient decision, or death. ORR was the sum of complete response (CR) and partial response (PR) rates. Duration of response (DoR) was calculated as the time from best response until disease progression or death from any cause in patients who achieved CR or PR. The best objective response, including CR, PR, stable disease (SD), and progressive disease (PD), was assessed locally at each participating center according to Response Evaluation Criteria in Solid Tumors v1.1 [17].

The secondary objective was to evaluate factors associated with survival outcomes in patients treated with EV, including treatment response, Eastern Cooperative Oncology Group performance status (ECOG PS), age, histological subtype (pure vs mixed UC), and primary tumor location (upper vs lower urinary tract). In addition, we examined factors potentially associated with the likelihood of achieving a response to EV (CR or PR), with particular focus on the influence of prior response to PEM (CR or PR) and the duration of prior PEM therapy (ToT_PEM_). The aim of these analyses was to assess both the association between prior PEM response and subsequent EV efficacy, and the potential impact of PEM treatment duration on EV outcomes.

### 2.3 Statistical analysis

DoR, OS, and ToT were analyzed as time-to-event outcomes using the Kaplan-Meier method. Median values and the corresponding 95% confidence interval (CI) were estimated. Patients without an event at the data cutoff were censored at the date of the last follow-up. The Kaplan-Meier method and log-rank test were used to compare OS and ToT. The median follow-up was estimated using the reverse Kaplan-Meier method from the start of EV therapy, with patients who had not experienced an event at the last follow-up being censored. Fisher's exact test was applied for pairwise comparisons of categorical variables, while chi-square tests were used for comparisons involving multiple categorical variables. Pearson's correlation coefficient was calculated to assess associations between continuous variables. To identify prognostic factors for OS and ToT, univariable Cox proportional-hazards regression analyses were first performed for all available clinical variables, including gender, age (continuous), smoking status (yes vs no), ECOG PS (≥2 vs 0-1), histology (pure UC vs variants), primary tumor site (bladder vs UTUC), metastatic disease at diagnosis (yes vs no), site of metastases (lymph node, lung, liver, bone, brain), and ToT_PEM_ (continuous), with prior response to PEM prespecified as the primary predictor of interest. Candidate variables for inclusion in the multivariable Cox models were selected on the basis of statistical significance in univariable analyses (*p* < 0.05) and/or established clinical relevance. Univariable results are reported only for the primary predictor of interest (prior PEM response), while other clinically relevant covariates are presented in the multivariable models as adjustment factors. Univariable and multivariable logistic regression analyses for models including all available clinical variables were performed to assess the impact of factors associated with objective response to EV (with CR/PR coded as response and SD/PD coded as nonresponse for the binary outcome). Candidate variables for the multivariable model were selected using the same approach as for the Cox models.

Statistical significance was set at a two-sided *p* value of <0.05. All analyses were conducted using MedCalc v19.6.4 (MedCalc Software, Mariakerke, Belgium).

### 2.4 Ethics approval

The study protocol was approved on September 28, 2023, by the ethics committee of the coordinating center (Marche Region, Italy; reference 2022 39/7875, ARON 2 study protocol, NCT05290038) and by the institutional review boards of all participating centers. The study was conducted in compliance with good clinical practice and the International Ethical Guidelines for Biomedical Research. The protocol was designed in accordance with the ethical principles outlined in the Declaration of Helsinki on human experimentation. Informed consent was obtained from all patients enrolled in the study.

## 3 Results

### 3.1 Study population

A total of 401 patients who received EV after PBC/PEM were included from the ARON-2EV data set (Supplementary Fig. 2). Median follow-up, estimated using the reverse Kaplan-Meier method, was 15.8 mo (95% CI 11.5–52.9). At data cutoff, 200 patients (50%) had died and 224 (56%) had experienced disease progression on EV. The median age at initiation of EV treatment was 69 yr (range 38–88). Of the patients, 303 (76%) were male. ECOG PS was 0–1 in 313 patients (78%) and ≥2 in 88 patients (22%). The most common metastatic sites were nonregional lymph nodes (61%) and the lungs (37%). Baseline clinical and demographic characteristics at initiation of EV are summarized in Table 1.

**Table 1:** Baseline clinical and demographic characteristics at initiation of enfortumab vedotin

Parameter	Result
Female, *n* (%)	98 (24)
Median age, yr (range)	69 (38–88)
Age ≥70 yr, *n* (%)	192 (48)
ECOG performance status, *n* (%)	
0–1	320 (80)
2	79 (19)
3	2 (1)
Current or former smoker, *n* (%)	263 (66)
Primary tumor location, *n* (%)	
Upper urinary tract	113 (28)
Lower urinary tract	288 (72)
Tumor histology, *n* (%)	
Pure urothelial carcinoma	291 (73)
Mixed histology	110 (27)
Timing of metastatic disease, *n* (%)	
Synchronous	111 (28)
Metachronous	290 (72)
Sites of metastasis, *n* (%)	
Nonregional lymph nodes	243 (61)
Lung	147 (37)
Liver	54 (13)
Bone	96 (24)
Brain	3 (1)

### 3.2 Survival outcomes and objective response

**Fig. 1:**
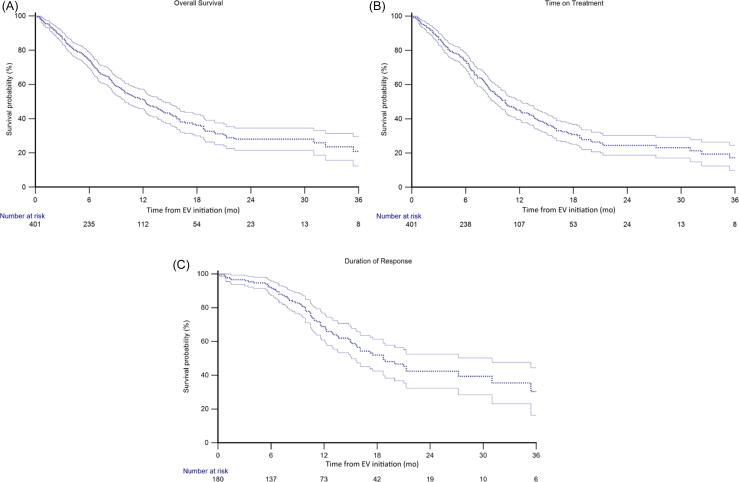
(A) Overall survival, (B) time on treatment, and (C) duration of response to enfortumab vedotin (EV) in the overall study population.

In the overall study population, median OS was 12.3 mo (95% CI 9.9–14.3; Fig. 1) and median ToT was 10.4 mo (95% CI 9.0–12.2). The best objective response was CR in 41 patients (10%), PR in 139 patients (35%), SD in 105 patients (26%), and PD in 116 patients (29%), resulting in an ORR of 45%. Among the 180 patients who achieved either CR or PR, median DoR was 18.7 mo (95% CI 15.1–31.0; Table 2 and Fig. 1).

**Table 2:** Distribution of responses to enfortumab vedotin according to prior response to pembrolizumab

Group	*n* (%)	CR, *n* (%)	PR, *n* (%)	SD, *n* (%)	PD, *n* (%)	ORR (%)	Median DoR, mo (95% CI)
Overall EV cohort	401	41 (10)	139 (35)	105 (26)	116 (29)	45	18.7 (15.1–31.0)
CR to pembrolizumab	19 (5)	5 (26)	8 (42)	5 (26)	1 (5)	68	–
PR to pembrolizumab	98 (24)	16 (16)	46 (47)	23 (23)	13 (13)	63	–
SD to pembrolizumab	96 (24)	10 (10)	27 (28)	30 (31)	29 (30)	38	–
PD to pembrolizumab	188 (47)	10 (5)	57 (30)	47 (25)	74 (40)	35	–

CI = confidence interval; CR = complete response; DoR = duration of response (for those achieving CR or PR); PR = partial response; ORR = overall response rate (CR + PR); PD = progressive disease; SD = stable disease.

### 3.3 Factors associated with survival outcomes

Median OS was significantly longer in the pooled group that achieved CR or PR (22.2 mo, 95% CI 18.7–38.1) than in the SD group (10.4 mo, 95% CI 8.8–47.3) and the PD group (4.0 mo, 95% CI 3.4–5.4; *p* < 0.001). Specifically, median OS was not reached (NR, 95% CI NR–NR) in the CR group and 18.7 mo (95% CI 15.0–31.0) in the PR group (Supplementary Fig. 3). The group with ECOG PS 0–1 had a significantly longer median OS than the group with ECOG PS ≥2 (14.1 mo, 95% CI 12.1–16.1 vs 6.0 mo, 95% CI 4.3–8.5; *p* < 0.001; Supplementary Fig. 3). There were no significant OS differences by age (hazard ratio [HR] 0.98, 95% 0.97−1.01; *p* = 0.06), histological subtype (median OS: pure UC 12.6 mo, 95% CI 10.0–15.1; mixed UC 10.9 mo, 95% CI 6.9–38.1; *p* = 0.4), or primary tumor site (median OS: upper tract 14.0 mo, 95% CI 9.9–32.3; lower tract 12.2 mo, 95% CI 9.5–14.1; *p* = 0.2).

### 3.4 Variables associated with objective response to EV

A statistically significant positive correlation was observed between the objective response to EV and prior CR/PR responses to PEM (correlation coefficient 0.237, 95% CI 0.15–0.34; *p* < 0.001), UC histology (correlation coefficient 0.167, 95% CI 0.009–0.208; *p* = 0.001), ECOG PS ≥2 (correlation coefficient −0.137, 95% CI −0.23 to 0.04; *p* = 0.007), and the presence of lung metastases (correlation coefficient −0.122, 95% CI −0.21 to 0.001; *p* = 0.02; Supplementary Fig. 4).

### 3.5 Impact of prior PEM duration and objective response on EV outcomes

Median ToT_PEM_ was 5.9 mo (95% CI 5.0–82.6). In the overall cohort, ToT_PEM_ was <6 mo in 216 patients (54%) and ≥6 mo in 185 patients (46%). Before receiving EV, 19 patients (5%) achieved a CR, 98 (24%) had a PR, 96 (24%) experienced SD, and 188 (47%) had PD on PEM. Median OS was 14.0 mo (95% CI 10.0–17.5) in the group with ToT_PEM_ ≥6 mo, compared to 10.5 mo (95% CI 8.6–47.3) in the group with ToT_PEM_ <6 mo (*p* = 0.05; Supplementary Fig. 5). Median ToT with EV was 12.1 mo (95% CI 9.3–14.0) in the group with ToT_PEM_ ≥6 mo, compared to 9.7 mo (95% CI 7.9–47.3) in the group with ToT_PEM_ <6 mo (*p* = 0.06; Supplementary Fig. 5).

**Fig. 2:**
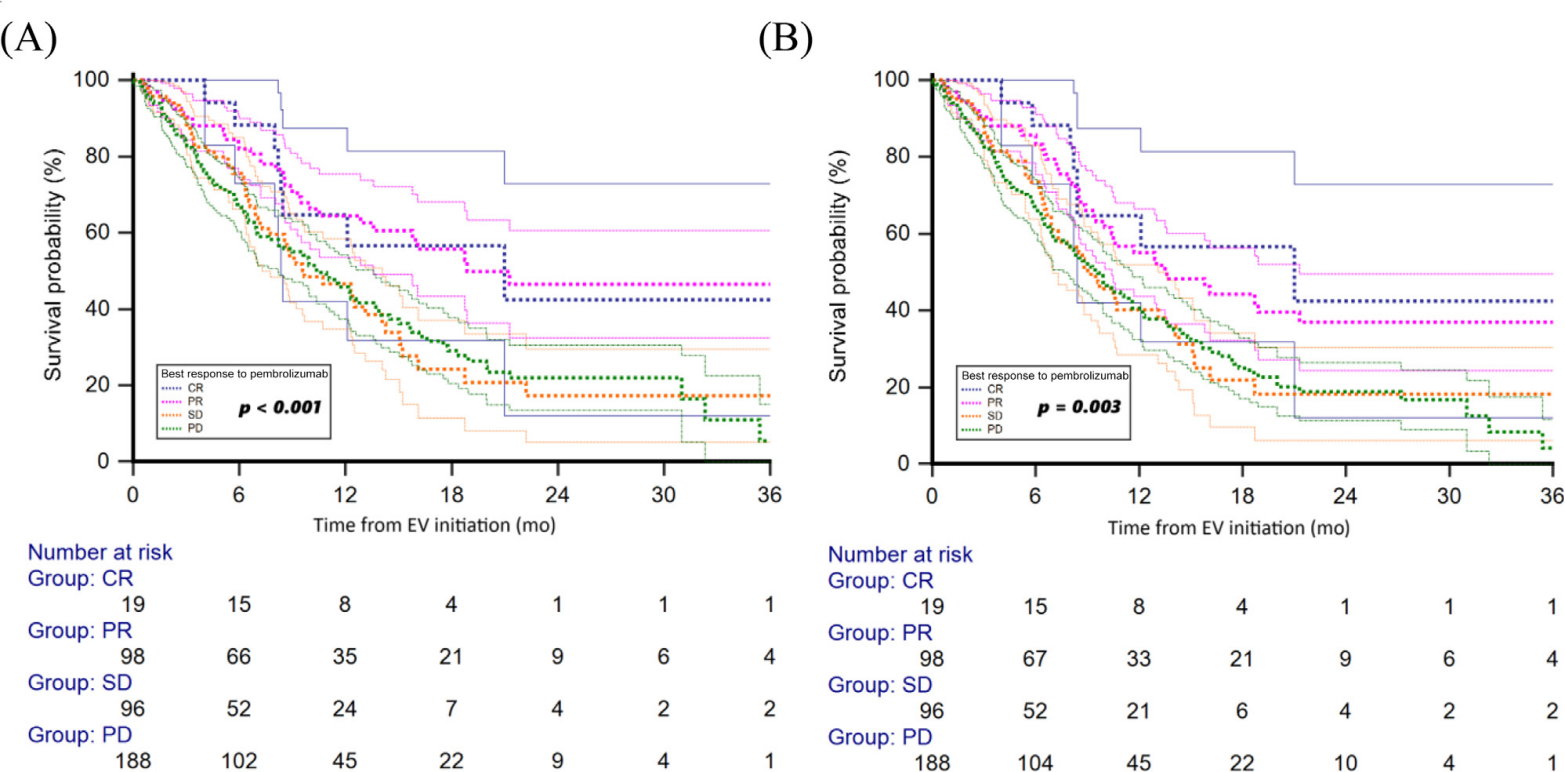
(A) Overall survival and (B) time on treatment with enfortumab vedotin (EV), stratified by best response to prior pembrolizumab treatment. CR = complete response; PR = partial response; SD = stable disease; PD = progressive disease.

OS varied significantly according to prior objective response to PEM: median was not reached (NR; 95% CI 8.2–NR) in the CR group, 18.9 mo (95% CI 13.6–38.1) in the PR group, 9.7 mo (95% CI 7.3–47.3) in the SD group, and 10.4 mo (95% CI 8.2–13.6) in the PD group (*p* < 0.001; Fig. 2). Median ToT on EV by prior response to PEM was NR (95% CI 8.2–NR) in the CR group, 13.6 mo (95% CI 10.0–21.3) in the PR group, 9.2 mo (95% CI 7.0–47.3) in the SD group, and 9.6 mo (95% CI 7.6–11.6) in the PD group (*p* < 0.001; Fig. 2).

Cox multivariable analysis results show that an objective response to prior PEM (CR/PR) remained an independent significant factor associated with both OS (HR 0.52, 95% CI 0.37−0.74; *p* < 0.001) and ToT (HR 0.58, 95% CI 0.42−0.79; *p* < 0.001; Table 3). Other independent factors significantly associated with OS were ECOG PS ≥2 (HR 1.87, 95% CI 1.34−2.61; *p* < 0.001), lung metastases (HR 1.49, 95% CI 1.12−1.98; *p* = 0.006), and liver metastases (HR 1.60, 95% CI 1.10–2.32; *p* = 0.01). Other independent factors associated with ToT were ECOG PS ≥2 (HR 1.89, 95% CI 1.37−2.60; *p* < 0.001) and liver metastases (HR 1.47, 95% CI 1.02–2.11; *p* = 0.04). When ToT_PEM_ was evaluated as a continuous variable in Cox models for OS (HR 0.99, 95% CI 0.98−1.01; *p* = 0.7) and ToT (HR 0.99, 95% CI 0.98−1.01; *p* = 0.3), no statistically significant associations were observed.

**Table 3:** Cox regression models for overall survival and time on treatment

Variable	Univariable		Multivariable	
	HR (95% CI)	*p* value	HR (95% CI)	*p* value
**Overall survival**				
Prior response to pembrolizumab (CR/PR vs SD/PD)	0.49 (0.35−0.69)	<0.001	0.52 (0.37−0.74)	<0.001
ECOG performance status (≥2 vs 0–1)	–	–	1.87 (1.34−2.61)	<0.001
Smoking status (yes vs no)	–	–	1.34 (0.98−1.82)	0.07
Lung metastases (yes vs no)	–	–	1.49 (1.12−1.98)	0.006
Liver metastases (yes vs no)	–	–	1.60 (1.10-2.32)	0.01
**Time on treatment**				
Prior response to pembrolizumab (CR/PR vs SD/PD)	0.55 (0.41−0.76)	<0.001	0.58 (0.42−0.79)	<0.001
ECOG performance status (≥2 vs 0–1)	–	–	1.89 (1.37−2.60)	<0.001
Lung metastases (yes vs no)	–	–	1.30 (0.99−1.70)	0.06
Liver metastases (yes vs no)	–	–	1.47 (1.02-2.11)	0.04

HR = hazard ratio; CI = confidence interval; ECOG = Eastern Cooperative Oncology Group; CR = complete response; PR = partial response; SD = stable disease; PD = progressive disease.

^a^ Univariable Cox regression analyses were performed for all clinical variables assessed, including sex, age (continuous), smoking status (yes vs no), ECOG performance status (≥2 vs 0–1), histology (pure urothelial carcinoma vs variants), primary tumor site (bladder vs upper tract), metastatic disease at diagnosis (yes vs no), site of metastases (lymph node, lung, liver, bone, brain), and time on treatment with pembrolizumab, with prior response to pembrolizumab prespecified as the primary predictor of interest. Univariable results are reported only for the primary predictor of interest (prior pembrolizumab response). Candidate variables for multivariable modeling were selected on the basis of statistical significance in univariable analyses and/or established clinical relevance. The final multivariable models therefore included ECOG performance status, smoking status, lung and liver metastases, and prior pembrolizumab response.

Multivariable logistic regression revealed that a prior objective response (CR/PR) to PEM was independently associated with an objective response (CR/PR) to EV (odds ratio [OR] 2.04, 95% CI 1.32–3.13; *p* = 0.001). Other clinical covariates were included as adjustment factors but are not reported, as they were beyond the scope of this analysis.

### 3.6 Association between response to prior PEM and subsequent EV activity

Among the 19 patients who achieved a CR to PEM, the response to subsequent EV was CR in five (26%), PR in eight (42%), SD in five (26%), and PD in one patient (5%). Among the 98 patients who achieved a PR to PEM, the response to subsequent EV was CR in 16 (16%), PR in 46 (47%), SD in 23 (23%), and PD in 13 (13%). Among the 96 patients who experienced SD to PEM, the response to subsequent EV was CR in ten (10%), PR in 27 (28%), SD in 30 (31%), and PD in 29 (30%). Finally, among 188 patients who were refractory to pembrolizumab (PD as best response), the response to subsequent EV was CR in ten (5%), PR in 57 (30%), SD in 47 (25%), and PD in 74 (40%), as shown in Table 2.

**Fig. 3:**
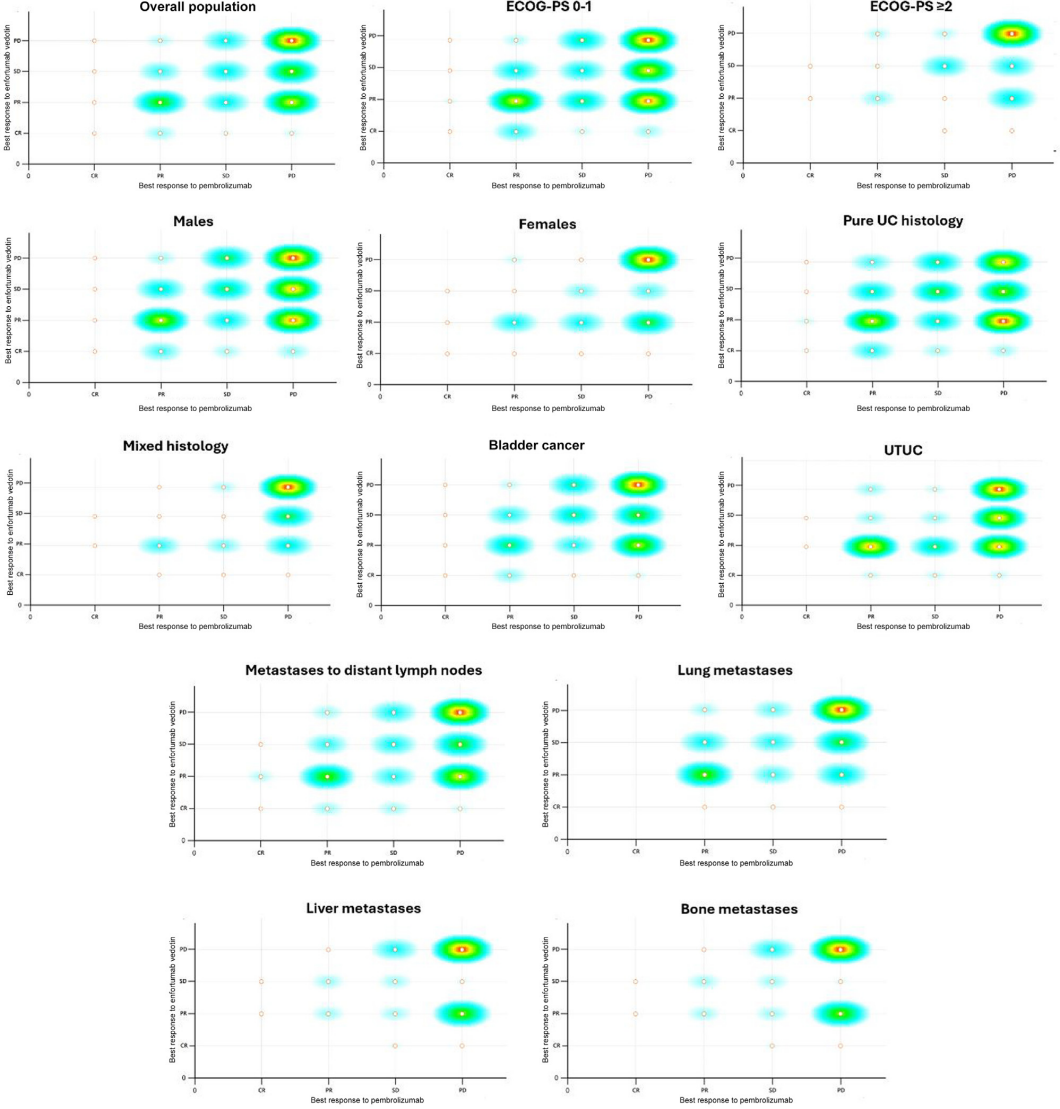
Scatter plots illustrating the correlation between objective responses to enfortumab vedotin and prior pembrolizumab in patients with metastatic urothelial carcinoma (UC), stratified by clinicopathological characteristics. ECOC-PS = Eastern Cooperative Oncology Group performance status; UTUC = upper tract UC.

Differences in the ORR (68% vs 63% vs 38% vs 35%; *p* < 0.001) and the rate of primary resistance (ie, PD) to EV (5% vs 13% vs 30% vs 40%; *p* < 0.001) across the four PEM response groups were statistically significant. The correlation between responses to EV and prior PEM stratified by clinicopathological features is illustrated in Fig. 3.

## 4 Discussion

As the treatment landscape for mUC continues to evolve, particularly with the introduction of EV plus PEM in the first-line setting [16], further research is needed to optimize treatment sequencing and identify potential predictive and prognostic factors useful in routine clinical practice [[18], [19], [20], [21]].

The present analysis of a subgroup from the ARON-2EV study provides valuable real-world evidence on the efficacy of EV in patients with mUC previously treated with PBC/PEM. We found median OS of 12.3 mo for patients treated with EV after PBC/PEM, which is comparable to the 12.9-mo median OS in EV-301. Similarly, the ORR of 45% in our study aligns with the 41% observed in EV-301 [15]. These findings suggest that the efficacy of EV is maintained in real-world settings, consistent with data from the previous clinical trials. However, there are several key differences between our study and the EV-301 clinical trial that should be noted. We included a broader patient population, in which 22% of patients had ECOG PS of ≥2, whereas EV-301 exclusively enrolled patients with ECOG PS 0–1 [15]. In addition, 27% of patients in our cohort had mixed UC histology, compared to 15% in EV-301 [15]. The inclusion of patients with poorer performance status and a higher proportion of patients with mixed histology types in our study better reflects real-world clinical practice. Furthermore, all the patients included in our analysis had received PEM before EV, whereas EV-301 allowed for prior treatment with any PD-1/PD-L1 inhibitor [15].

In the context of personalized oncology, particularly in UC, the significance of predictive and prognostic factors is becoming more evident. NECTIN4 has recently shown promise as a molecular predictive biomarker for EV response. Available evidence indicates that high NECTIN4 expression and gene amplification are frequently co-occurring features in mUC and are both associated with better clinical outcomes with EV [22,23]. We observed a significant association between EV activity and outcomes with prior PEM. In exploratory correlation analysis, prior CR/PR to PEM was significantly associated with achieving a CR/PR to EV (*p* < 0.001). In addition, multivariable logistic regression confirmed that prior response to PEM was independently associated with a response to EV (OR 2.04, 95% CI 1.32–3.13; *p* = 0.001), which further supports our findings. Cox multivariable analysis showed that CR/PR to prior PEM remained an independent significant factor associated with superior OS (HR 0.52, 95% CI 0.37–0.74; *p* < 0.001) and superior ToT (HR 0.58, 95% CI 0.42–0.79; *p* < 0.001).

In the initial exploratory analysis, ToT_PEM_ was dichotomized at 6 mo (the cohort median), and longer duration of prior PEM treatment was associated with significantly longer OS (14.3 vs 10.5 mo; *p* = 0.03). However, when ToT_PEM_ was subsequently evaluated as a continuous variable in Cox models for OS and ToT, no statistically significant associations were observed. This discrepancy suggests that the effect observed may reflect a threshold phenomenon rather than a linear relationship, and should therefore be interpreted with caution given the retrospective and exploratory nature of the analysis.

Interestingly, we found that best response to prior PEM was associated with a greater benefit for the OS outcome than for ToT. One potential explanation is that patients with a response to prior PEM may have more favorable tumor biology that leads to prolonged OS regardless of time on EV. In addition, ToT can be influenced by factors beyond efficacy, including toxicity and logistical considerations.

Notably, our study results suggest that the prior immunotherapy outcome may serve as a surrogate predictor of EV effectiveness. This aligns with observations from KEYNOTE-045, in which longer exposure and a durable response to PEM were associated with better OS [24]. Although the impact of PEM response on outcomes with subsequent therapies was not specifically addressed in that trial, the present data suggest that such a prior benefit may be associated with enhanced efficacy of later treatments such as EV. These findings also raise the possibility of a biologically relevant interaction between ICIs and subsequent ADC therapy. One hypothesis is that PEM may prime the tumor microenvironment and enhance its susceptibility to later cytotoxic treatments by increasing T-cell infiltration, altering cytokine profiles, or promoting an immunostimulatory milieu [25,26]. In addition, it has been shown that EV induces immunogenic cell death, which may synergize with the residual immune activation induced by PEM [27]. However, our findings could also be influenced by selection bias or confounding factors. Importantly, our results demonstrate that even the group whose best response to prior PEM was PD achieved an ORR of 35% with EV, which underscores the general value of EV in this difficult-to-treat population.

The evolving treatment landscape for mUC introduces additional complexity in treatment sequencing. Current guidelines recommend EV plus PEM as a preferred first-line option alongside chemotherapy plus immunotherapy combination in the cisplatin-eligible patient population [6]. The role of avelumab in switch maintenance after first-line chemotherapy further expands therapy sequencing options. In a previous analysis of the ARON-2EV study, we focused specifically on the population with prior PBC followed by avelumab maintenance. Interestingly, median OS was 12.7 mo, which is comparable to results in the present study, even though the two analyses focused on different patient populations: avelumab was administered to patients who responded to first-line PBC, whereas PEM was given to those with platinum-refractory disease [28]. Similar findings were observed in another real-world study in which EV was administered after chemotherapy followed by avelumab switch maintenance, with median OS of 11.6 mo [29]. In addition, results from the AVENANCE study revealed median OS of 40.8 mo with first-line chemotherapy when ADCs were used following avelumab maintenance [30], which reinforces the added survival benefit of ADCs such as EV in this setting.

Despite its strengths, the ARON-2EV study has several limitations. Its retrospective design may introduce selection bias, and the absence of centralized radiology review could lead to variability in response assessment. The heterogeneity of the patient population and the lack of a control arm further complicate the interpretation of outcomes, while the absence of NECTIN4 expression data limits the ability to perform biomarker-based analyses. In addition, detailed information on EV dose adjustments was not consistently available for the present analysis. Furthermore, the exact cause of death was not systematically recorded, which limited our ability to apply competing-risks methods to distinguish death from other reasons for treatment discontinuation in analyses of ToT and ToT_PEM_. As in prior pivotal and real-world EV studies, death was included as part of the composite event definition for ToT. We acknowledge that this methodological choice may overestimate the risk of treatment discontinuation in the presence of competing events, which represents a limitation of our analysis.

Despite these limitations, ARON-2EV makes a unique contribution to our understanding of EV efficacy in a real-world setting. Unlike clinical trials, the study includes a broader and more diverse patient population, including individuals with ECOG performance status ≥2 and a higher proportion of cases with mixed UC histology. It also underscores the increasing complexity of treatment sequencing in mUC, particularly in the context of immunotherapy and ADCs. Our findings add to the growing body of real-world evidence suggesting that prior response to ICIs may influence subsequent outcomes with EV, a relationship that remains incompletely understood. The potential clinical relevance of this observation lies in its potential to inform more personalized treatment strategies, guided by patients’ prior therapeutic exposure and tumor behavior. The EV activity demonstrated in pembrolizumab-refractory patients further supports its role in a particularly challenging treatment population.

In spite of the contributions of our study, several critical knowledge gaps persist. First, the biological mechanisms underpinning the interaction between immune modulation and ADC efficacy are not yet well elucidated. Second, prospective data are limited regarding optimal treatment sequencing and the validation of predictive and prognostic biomarkers for EV effectiveness. Bridging these gaps will require integrated translational research that links clinical outcomes to molecular profiling and immune landscape characterization. Looking ahead, we anticipate that the coming years will bring continued refinement of therapeutic strategies in mUC, with increasing emphasis on biomarker-driven decision-making and rational combinations of ICIs, targeted agents, and ADCs. The development of next-generation ADCs, greater use of liquid biopsies, and broader integration of real-world data into research and clinical practice are likely to play pivotal roles in shaping individualized treatment approaches. The present study offers a valuable real-world perspective that complements existing clinical trial data and highlights the importance of ongoing investigations into how prior therapies influence the efficacy of subsequent treatments.

## 5 Conclusions

In conclusion, our study results support the real-world efficacy of EV in patients with mUC previously treated with PBC followed by PEM. The findings largely align with those from the EV-301 trial and offer additional insights into treatment sequencing and outcomes in a broader patient population.

  ***Author contributions***: Ondrej Fiala had full access to all the data in the study and takes responsibility for the integrity of the data and the accuracy of the data analysis.

  *Study concept and design*: Santoni.

*Acquisition of data*: Fiala, Grillone, Fujita, Grande, Giannatempo, Taha, Myint, Büttner, Gómez de Liaño, Kanesvaran, Facchini, Yano, Formisano, Poprach, Conteduca, Pirshtuk, Studentova, Kopecky, Sammarco, Mota, Incorvaia, Nasso, Maffezzoli, Ghose, Soares, Buti, Marques Monteiro, Massari, Gupta, Bellmunt, Banna, Santoni.

*Analysis and interpretation of data*: Santoni.

*Drafting of the manuscript*: Fiala, Banna, Santoni.

*Critical revision of the manuscript for important intellectual content*: Santoni, Fiala.

*Statistical analysis*: Santoni.

*Obtaining funding*: None.

*Administrative, technical, or material support*: Fiala.

*Supervision*: None.

*Other*: None.

  ***Financial disclosures:*** Ondrej Fiala certifies that all conflicts of interest, including specific financial interests and relationships and affiliations relevant to the subject matter or materials discussed in the manuscript (eg, employment/affiliation, grants or funding, consultancies, honoraria, stock ownership or options, expert testimony, royalties, or patents filed, received, or pending), are the following: Ondrej Fiala reports honoraria from Novartis, Janssen, Merck, Ipsen, BMS, MSD, Pierre Fabre, and Pfizer for consultations and lectures unrelated to this work. Francesco Grillone reports a consultant/advisory board role for Astellas, Merck, MSD, Recordati, Ipsen, and Bayer; and speaker honoraria or travel support from Astellas, Johnson & Johnson, Ipsen, Bayer, Novartis, Merck, AstraZeneca, Recordati, and Takeda, all unrelated to this work. Enrique Grande reports honoraria for advisory board roles, meetings, and/or lectures from Pfizer, BMS, IPSEN, Roche, Eisai, Eusa Pharma, MSD, Sanofi, AAA, Novartis, Pierre Fabre, Lexicon, and Celgene; and unrestricted research grants from Pfizer, AstraZeneca, MTEM/Threshold, Roche, Ipsen, and Lexicon. Tarek Taha reports institutional support for attending meetings and/or travel from Pfizer, and honoraria from BMS, MSD, Merck Serono, and Pfizer. Thomas Büttner reports honoraria and/or research support from Astellas, Ipsen, MSD, and Merck, all unrelated to this work. Alfonso Gómez de Liaño reports honoraria for advisory board roles, consultations, or educational events from AAA HealthCare, Astellas, AstraZeneca, Bayer, BMS, Ipsen, Johnson & Johnson, MSD, Merck KGaA, Novartis, Recordati Rare Diseases, and Roche; and institutional research funding from AstraZeneca, Bicycle Therapeutics, Genmab A/S, Gilead Sciences, Johnson & Johnson, MedSIR, Merck KGaA, MSD, Pfizer, Roche, and Syneos Health, all unrelated to this work. Gaetano Facchini reports consultant/advisory board roles for and speaker honoraria or travel support from Astellas, Johnson & Johnson, AstraZeneca, Recordati, Ipsen, Bayer, Pfizer, Novartis, and MSD unrelated to the present work. Akihiro Yano reports speaker/advisory honoraria from Janssen, Bayer, Pfizer, Astellas, Takeda, Eisai, MSD, Bristol-Myers Squibb, Merck, and Kissei unrelated to the present work. Alexandr Poprach reports payments or honoraria for lectures, presentations, speaker bureaus, manuscript writing, or educational events from BMS, Ipsen, Roche, Astellas, Merck, Eisai, MSD, Novartis, and Pfizer unrelated to the present work. Vincenza Conteduca reports a consultant/advisory board role for Johnson & Johnson, Astellas, Merck, AstraZeneca, Amgen, Eisai, Recordati, Novartis, Ipsen, and Bayer; and speaker honoraria or travel support from Astellas, Johnson & Johnson, Ipsen, Bayer, Gilead, Novartis, and Bristol-Myers Squibb unrelated to the present work. Alina Pirshtuk reports speaker honoraria or travel support from Merck, AstraZeneca, Servier, and BMS outside the present work. Hana Studentova reports payments or honoraria for lectures, presentations, or speaker bureaus from BMS, Ipsen, Astellas, Merck, MSD, Novartis, and Pfizer unrelated to the present work. Cecilia Nasso reports honoraria for meetings and/or speaking at scientific events from BMS, Ipsen, Eisai, MSD, and Astellas unrelated to the present work. Andrey Soares reports honoraria from Janssen, Pfizer, Bayer, Merck Serono, and Novartis; consulting or advisory fees from Janssen, Bayer, AstraZeneca, MSD, Pfizer, and Novartis; institutional research funding from Bristol-Myers Squibb, Astellas, and AstraZeneca; travel and accommodation expenses from Bayer, Janssen, MSD, Merck Serono, and Adium Pharma; and ownership interests in Brazilian Information Oncology, all unrelated to the present work. Sebastiano Buti reports honoraria for speaker and advisory roles from AstraZeneca, Bristol-Myers Squibb, Ipsen, Merck, Eisai, MSD, Novartis, Gentili, Astellas, and Pfizer; and research funding from Novartis and Pfizer. Augusto Mota reports research support, honoraria, and/or travel and accommodation expenses from AstraZeneca, Bayer, BMS, Janssen, Ipsen, Merck, Astellas, and Pfizer, all unrelated to the present work. Fernando Sabino Marques Monteiro reports research support from Merck Sharp & Dohme and Foundation Medicine; honoraria from Janssen, Ipsen, Bristol-Myers Squibb, and Merck Sharp & Dohme; travel expenses from Novartis, Bayer, Adium Pharma, Merck, and Merck Sharp & Dohme; and ownership interests in Brazilian Information Oncology, all unrelated to the present work. Francesco Massari reports research support and/or honoraria from Advanced Accelerator Applications, Astellas, AstraZeneca, Bayer, BMS, Janssen, Ipsen, Merck, MSD, and Pfizer outside the present work. Giuseppe Luigi Banna reports personal fees from advisory board roles for Accord, AstraZeneca, Amgen, and Merck; speaker bureau participation for Astellas, AstraZeneca, Amgen, Bayer, Merck, and Pfizer; interests in four patents held by ST Microelectronics; and travel and accommodation expenses from Accord, Merck, and Janssen. Matteo Santoni reports support and honoraria from Janssen, Bristol-Myers Squibb, Ipsen, MSD, Astellas, and Bayer, all unrelated to the present work. The remaining authors have nothing to disclose.

  ***Funding/Support and role of the sponsor*:** None.
