# Supplementary material for: Efficacy of Enfortumab Vedotin After Platinum Chemotherapy and Pembrolizumab in Metastatic Urothelial Carcinoma: A Multicenter Real-World Analysis of the ARON-2EV Cohort
Source: Eur Urol Open Sci. 2025 Nov 3;82:111–20. doi: 10.1016/j.euros.2025.10.010 (PMC12630326; doi:10.1016/j.euros.2025.10.010)
Supplement: Supplementary Data 1 [file mmc1.docx]

**Supplementary Material**

**
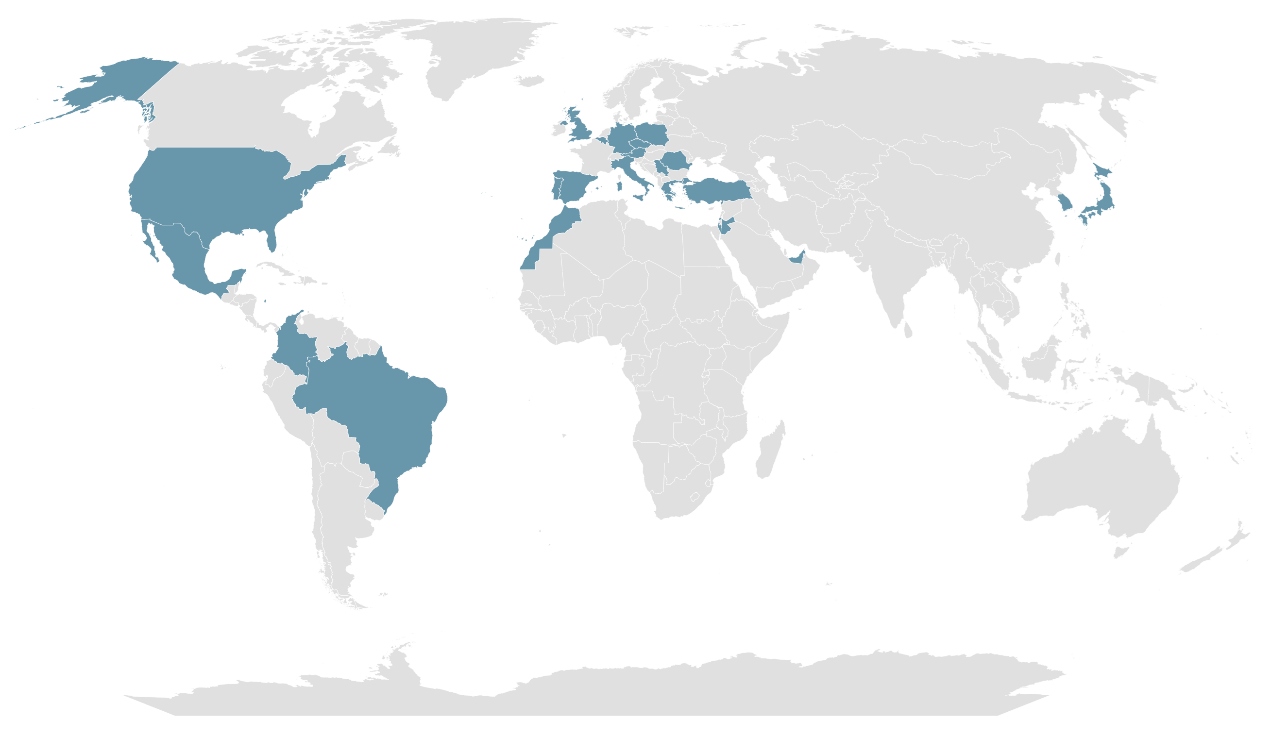
**

**Supplementary Figure 1.** Map of countries participating in the ARON-2EV Study


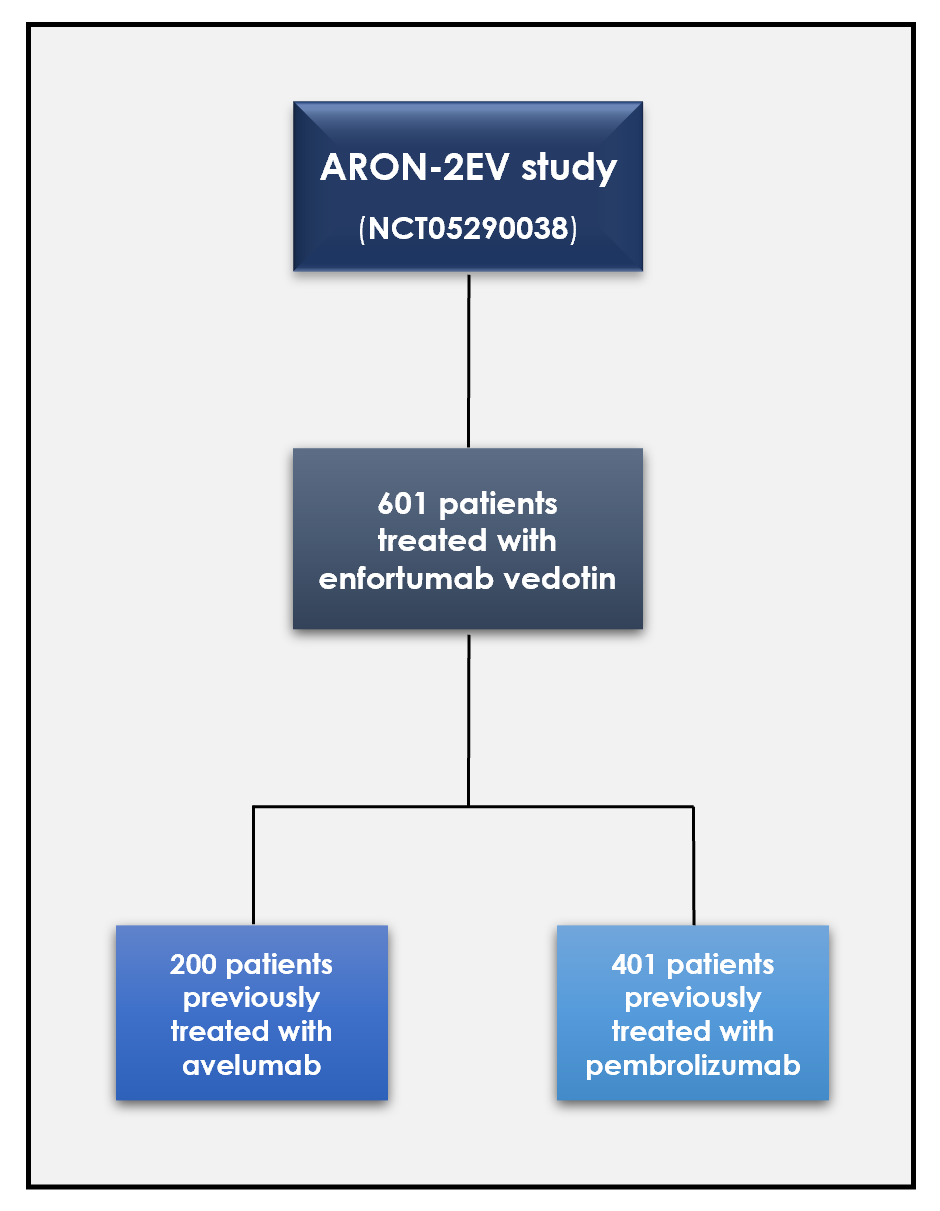


**Supplementary Figure 2**. Selection process from the ARON-2EV dataset


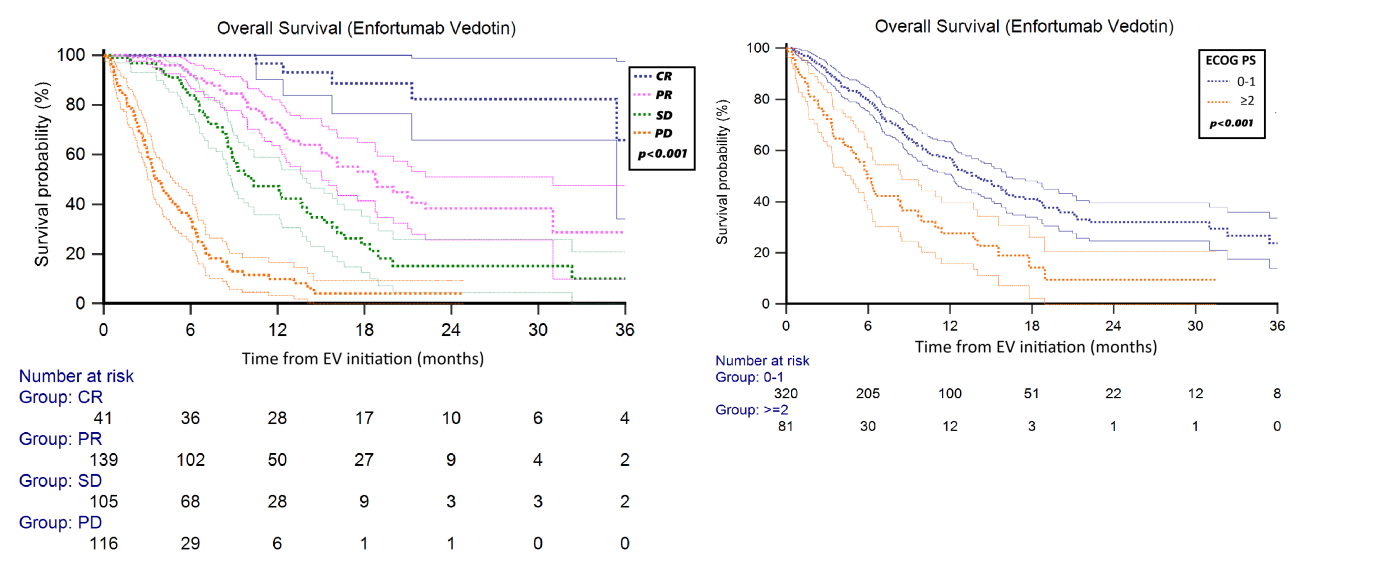


**Supplementary Figure 3.** Subgroup analyses of Overall Survival in patients receiving Enfortumab Vedotin (EV)

**
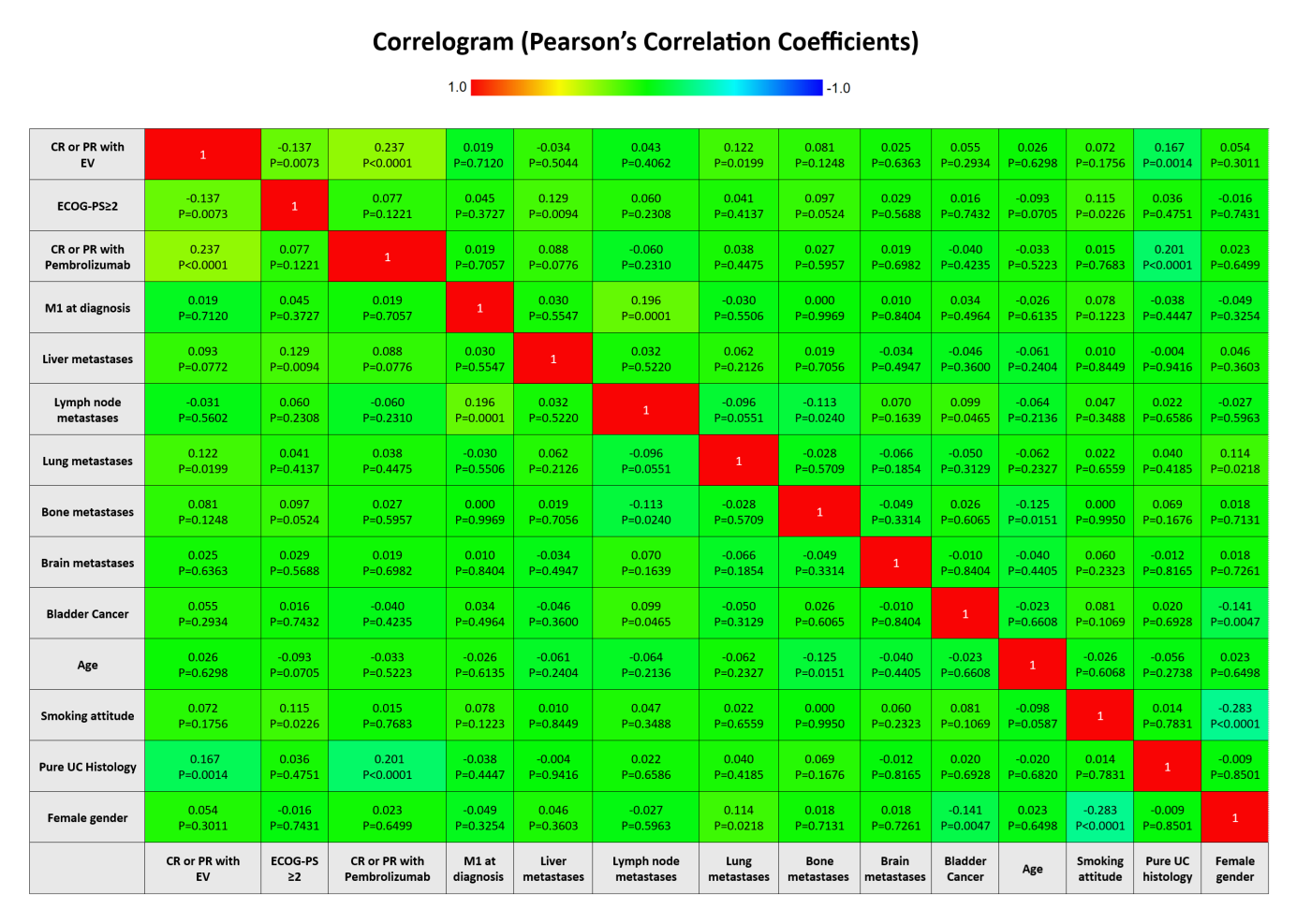
**

**Supplementary Figure 4.** Correlogram of Pearson’s correlation coefficients showing factors associated with objective response to enfortumab vedotin (EV)


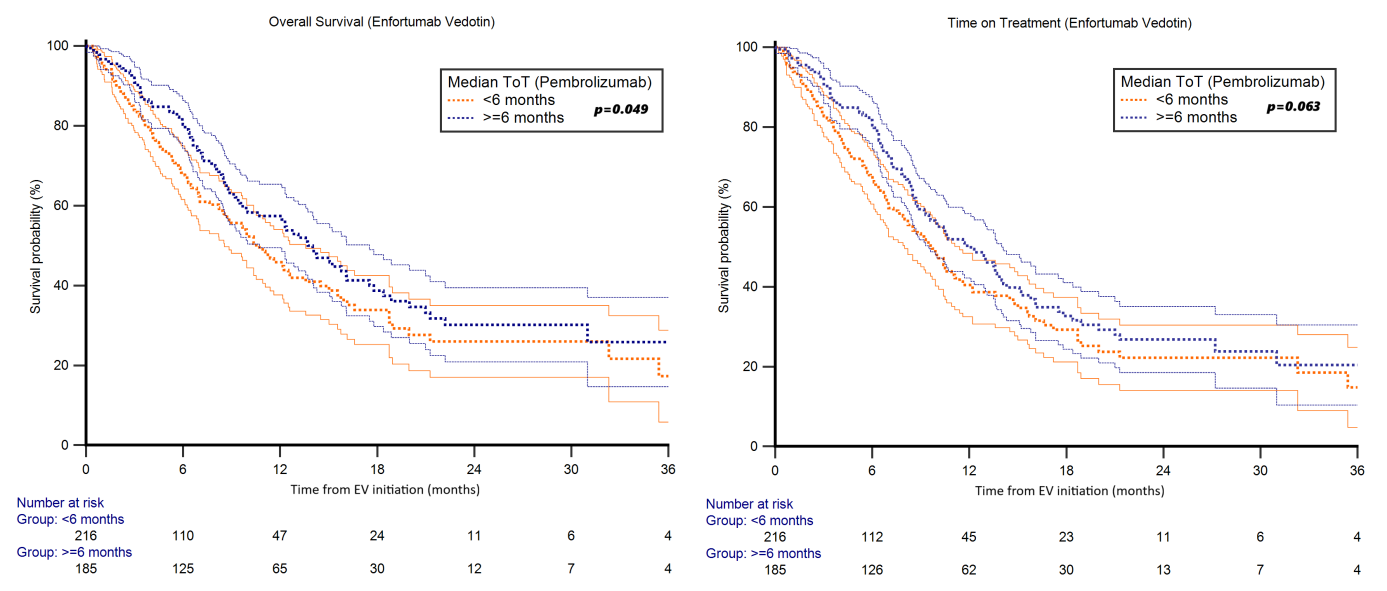


**Supplementary Figure 5.** Overall Survival (OS) and Time on Treatment (ToT) from the start of enfortumab vedotin (EV) therapy in patients stratified by the ToT with prior pembrolizumab (ToT_PEM_)
